# Cardiology fellows-in-training are exposed to relatively high levels of radiation in the cath lab compared with staff interventional cardiologists—insights from the RECAP trial

**DOI:** 10.1007/s12471-019-1254-1

**Published:** 2019-03-06

**Authors:** W. Vlastra, B. E. Claessen, M. A. Beijk, K. D. Sjauw, G. J. Streekstra, J. J. Wykrzykowska, M. M. Vis, K. T. Koch, R. J. de Winter, J. J. Piek, J. P. S. Henriques, R. Delewi

**Affiliations:** 1 Heart Center, Department of Clinical and Experimental Cardiology, Amsterdam UMC, University of Amsterdam, Amsterdam, The Netherlands; 2 Department of Biomedical Engineering and Physics, Amsterdam UMC, University of Amsterdam, Amsterdam, The Netherlands

**Keywords:** Radiation exposure, Radiation protection, Interventional cardiologist, Cardiology fellows-in-training

## Abstract

**Background:**

Interventional cardiologists are inevitably exposed to low-dose radiation, and consequently are at risk for radiation induced diseases like cataract and left-sided brain tumours. Operator behaviour may possibly be the largest influencer on radiation exposure. We hypothesised that awareness regarding radiation exposure grows as skill and the general experience in the catheterization laboratory increase.

**Objectives:**

In this study we determined the difference in the relative radiation exposure of staff interventional cardiologists compared with cardiology fellows-in-training.

**Methods:**

During this prospective trial the operator’s radiation exposure (E in µSv) was measured at chest height during 766 diagnostic catheterisations and percutaneous coronary interventions. Also, the patient exposure (DAP in mGy·cm^2^), representing the amount of radiation administered by the operator per procedure, was collected. The primary outcome of this study was the difference in relative exposure between staff interventional cardiologists versus cardiology fellows-in-training (E/DAP).

**Results:**

From January to May 2017, staff interventional cardiologists performed 637 procedures and cardiology fellows-in-training 129 procedures. The performance of relatively complex procedures by staff interventional cardiologists resulted in a 74% higher use of radiation compared with fellows-in-training. Consequently, staff interventional cardiologists were exposed to 50% higher levels of actual radiation exposure. However, when correcting for the complexity of the procedure, by comparing the relative operator exposure (E/DAP), fellows-in-training were exposed to a 34% higher relative exposure compared with staff interventional cardiologists (*p* = 0.025).

**Conclusions:**

In the current study, when corrected for complexity, cardiology fellows-in-training were exposed to significantly higher radiation levels than staff interventional cardiologists during catheterisation procedures.

## What’s new?

- Since staff interventional cardiologists perform more complex procedures in the catheterisation laboratory compared with cardiology fellows-in-training, they use 74% higher radiation levels (dose area product—DAP), consequently staff interventional cardiologists were exposed to 50% higher levels of actual radiation exposure (E in µSv, measured at chest height).
- However, after correcting the actual radiation exposure for the amount of used radiation (E/DAP), fellows-in-training were exposed to 34% higher relative radiation exposure levels compared with staff interventional cardiologists.
- These findings highlight that there should be more attention for radiation safety behaviour during the training of cardiology fellows.

## Introduction

Despite the technological developments of the last decades, the profession of an interventional cardiologist is inevitably related to exposure to low-dose radiation. Consequently, interventional cardiologists are at risk for developing radiation-induced diseases such as cataract and left-sided brain tumours [1, 2]. The operator’s radiation exposure strongly varies per procedure, depending on patient characteristics, complexity of the performed procedure, and radiation protection equipment [3]. However, operator behaviour may possibly be the largest influencer of radiation exposure. We hypothesised that awareness regarding radiation exposure among operators grows as skill and the general experience in the catheterisation laboratory increase. Accordingly, we aimed to investigate the difference in the relative radiation exposure of cardiology fellows-in-training compared with staff interventional cardiologists.

## Methods

This is a sub-study of the RECAP trial (NCT03139968), a double-blind, sham-controlled, randomised clinical trial, which evaluated the efficacy of a radiation-absorbing drape (the RADPAD 5100A-O, Worldwide Innovations & Technologies, Inc., Lenexa, Kansas, USA) [4]. In this single-centre prospective, all-comer trial, performed between January and May 2017, the real-time operator’s radiation exposure (E in µSv) was measured at chest height with a dosimeter (PDM, DoseAware, Philips Medical Systems, the Netherlands) during 766 diagnostic catheterisations (coronary angiography) and percutaneous coronary interventions (PCI). The patient exposure, representing the amount of radiation administered by the operator per procedure, was also collected (dose area product [DAP] in mGy·cm^2^). The primary endpoint of the current sub-analysis was the difference in relative radiation exposure between cardiology fellows-in-training and staff interventional cardiologists. This was defined as the ratio between the primary operator’s radiation exposure (E), and patient’s radiation exposure (DAP), both measured per procedure. The relative radiation exposure (E/DAP), rather than the actual radiation exposure, was chosen in order to correct for the inter-procedural variance in the administered amount of radiation [5]. This way it is possible to test the actual operator behaviour rather than the complexity of a procedure. Since interventional procedures are often performed by two operators, the primary operator was defined as the operator that had the closest location to the radiation source, and accordingly received the highest radiation exposure. The study protocol was approved by the Institutional Review Boards of the Amsterdam University Medical Center. Since the current study does not impose interventions on the patient and has no risks or benefits for the patient, informed consent was not required.

## Results

**Fig. 1:**
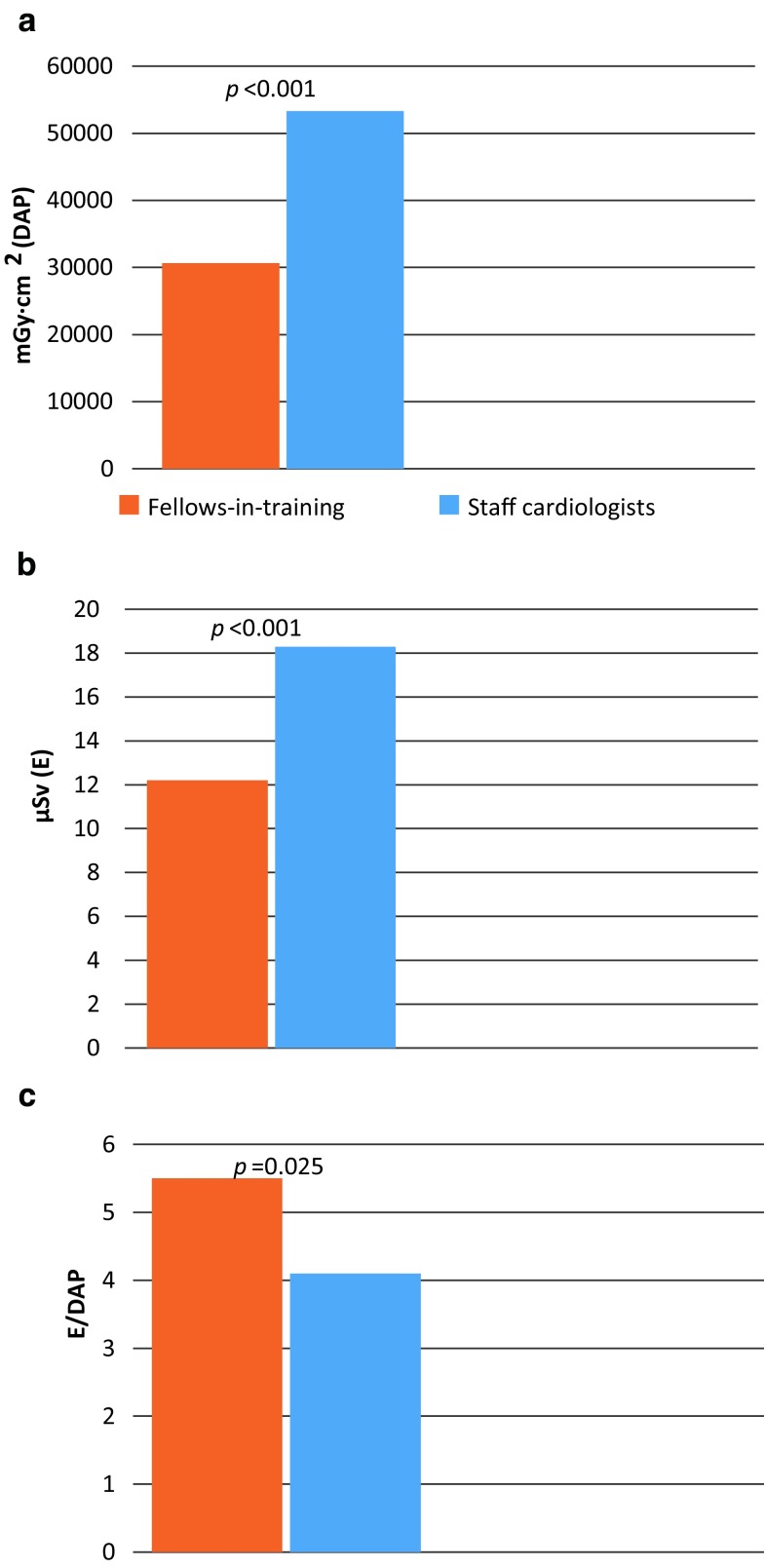
Operator’s radiation use and operator radiation exposure. Relative operator exposure (**c**) (×10^−4^) is defined as the ratio between the actual exposure of the primary operator at chest level (**b**) and the operator’s radiation use (**a**) per procedure and therefore corrects for the complexity of the procedure. (*DAP* dose area product)

During the 4‑month study period of the RECAP trial, staff interventional cardiologists were the primary operator during 637 coronary procedures, whereas cardiology fellows-in-training were the primary operator in 129 procedures. The mean patient age was 67 ± 11 years, 70% was male and the mean body mass index (BMI) was 27.7 ± 5.0. Most patient characteristics, including BMI, were comparable among patients treated by staff cardiologists or fellows-in-training (Tab. 1). As expected, staff interventional cardiologists more often performed relatively complex procedures compared with cardiology fellows-in-training. There was a trend towards a higher number of patients presenting with STEMI treated by staff cardiologists compared with cardiology fellows-in-training (8% vs 4%, *p* = 0.08). Also, staff interventional cardiologists more frequently performed PCI compared with cardiology fellows-in-training (55% vs 35%, *P* < 0.001). Additionally, interventional cardiologists more frequently performed PCI of chronic total occlusions (CTO) (5% vs 1%, *p* = 0.02). The performance of these relatively complex procedures by staff interventional cardiologists resulted in a 74% higher use of radiation compared with fellows-in-training (DAP, Fig. 1a). Consequently, staff interventional cardiologists were exposed to 50% higher levels of actual radiation exposure compared with cardiology fellows-in-training (E, Fig. 1b). However, when correcting for the complexity of the procedure, by evaluating the relative operator exposure (E/DAP), fellows-in-training were exposed to a 34% higher relative exposure compared with staff interventional cardiologists (Fig. 1c, *p* = 0.03).

**Table 1:** Patient and procedural characteristics

	staff cardiologists	fellows-in-training	*p*-value
	(*n* = 637)	(*n* = 129)	
*demographics*			
age (years)	67 ± 12	67 ± 10	0.48
male gender	440 (69)	92 (71)	0.61
*medical history*			
BMI	27.6 ± 5.0	28.0 ± 4.9	0.44
previous myocardial infarction	169 (27)	25 (20)	0.08
previous PCI	187 (30)	35 (27)	0.60
previous bypass surgery	55 (9)	11 (9)	0.98
*risk factors*			
diabetes mellitus	156 (25)	30 (24)	0.73
known hypertension	367 (60)	63 (50)	0.04
family history of CAD	322 (56)	68 (56)	0.99
hypercholesterolaemia	282 (48)	48 (39)	0.06
current cigarette smoking	122 (21)	26 (21)	0.99
*presentation*			
STEMI	53 (8)	5 (4)	0.08
NSTEMI/UA	124 (20)	22 (17)	0.53
stable CAD	366 (58)	79 (61)	0.43
other^a^	94 (15)	23 (18)	0.38
*procedural characteristics*			
radial access	497 (78)	109 (85)	0.10
PCI	349 (55)	45 (35)	<0.001
lesions treated per PCI	1.44 ± 0.67	1.26 ± 0.50	0.12
location of treated lesion: LM or LAD	162 (54)	22 (58)	0.61
location of treated lesion: CX	90 (30)	9 (24)	0.44
location of treated lesion: RCA	105 (35)	10 (26)	0.31
PCI of chronic total occlusion	34 (5)	1 (1)	0.02
iFR/FFR only^b^	46 (7)	15 (12)	0.09
Skin-to-Skin time (min)	47.1 ± 27.0	45.5 ± 21.8	0.55

Data are number (%) or mean ± SD

*BMI* body mass index, *PCI* percutaneous coronary intervention, *CAD* coronary artery disease, *STEMI* ST-elevation myocardial infarction, *NSTEMI* non-ST-elevation myocardial infarction, *UA* unstable angina, *LM* left main coronary artery, *LAD* left anterior descending coronary artery, *CX* circumflex artery, *RCA* right coronary artery, *iFR/FFR* instantaneous wave-free ratio/fractional flow reserve

^a^Heart failure, pre transplantation, pre-valve replacement

^b^Without PCI

## Conclusions

In conclusion, in the current study cardiology fellows-in-training were exposed to significantly higher radiation levels than staff interventional cardiologists during catheterisation procedures, when corrected for complexity. We hypothesise that cardiology fellows-in-training are exposed to higher radiation exposure than staff interventional cardiologists for two reasons. Firstly, fellows-in-training, in their eagerness to learn, are often preoccupied with their patient’s health, potentially forgetting to protect their own health by maintaining appropriate distance from the scatter radiation source during the moments of fluoroscopy, and forgetting to optimise collimation settings. Secondly, we hypothesise that fellows-in-training, more than their mentors feel the need to make additional recordings to secure themselves of a successful procedure, increasing total fluoroscopy time. In the current study there was a trend to fellows-in-training more frequently performing procedures through the radial artery compared with staff cardiologists (85% vs 78%, *p* = 0.10). The performance of more radial procedures could have reduced the distance between the fellow-in-training and the radiation source, increasing the radiation exposure. However, in the current study radial artery access was not related to a higher relative radiation exposure (*p* = 0.16). Moreover, during radial procedures, the left radial artery was used in a minority of the procedures, both by cardiology fellows-in-training and staff interventional cardiologists (8% vs 11%, *p* = 0.45).

There are several strategies to reduce the radiation exposure of cardiology fellows-in-training. Firstly, the total radiation use can be reduced using modern X‑ray systems. In accordance with the ALARA principle (As Low As Reasonably Achievable), the appropriate ratio between optimal image quality and radiation dose is optimised using modern X‑ray systems that combine real-time image noise reduction algorithms with shorter X‑ray pulses and smaller focal spots. The procedures in the current study were performed using multiple catheterisation rooms equipped with either the newer Philips AlluraClarity FD10 (including noise reduction technology) or the Philips Allura Xper FD10 (Philips Medical Systems, the Netherlands). In the current analysis we corrected for the use of these two different systems. Interestingly, the radiation use was 65% lower during diagnostic angiograms performed using the AlluraClarity X‑ray system. Likewise, the operator radiation exposure was 63% lower (both *p* < 0.001). These findings are in accordance with an earlier study that showed a 50% reduction of the operator exposure, and importantly without a reduction in image quality [6]. Likewise, additional shielding measures can be used to reduce the radiation exposure of fellows-in-training. Radiation-absorbing drapes reduce radiation exposure with 20% [4], leaded glasses reduce scatter radiation to the eye with a factor of 5 to 10 [7], but are often reported as being uncomfortable [8]. Moreover, protective caps considerably reduce cranial radiation exposure [9].

Nevertheless, the higher radiation exposure of fellows-in-training in the current study was the consequence of operator behaviour and therefore could and should be adjusted. We strongly recommend that during training of cardiology fellows, there should be more attention for radiation safety behaviour, this includes appropriate distance from the scatter radiation source during fluoroscopy. In a randomised controlled trial the use of real-time dosimetry considerably reduced the operator’s radiation exposure [10], accordingly real-time dosimeters could provide aid during the training of cardiology fellows. Cardiology fellows-in-training should not only be evaluated by their seniors on their PCI successes but also on their radiation safety behaviour. Because if the mentors won’t protect their pupil’s health, who will?
